# Reiterative infusions of MSCs improve pediatric osteogenesis imperfecta eliciting a pro‐osteogenic paracrine response: TERCELOI clinical trial

**DOI:** 10.1002/ctm2.265

**Published:** 2021-01-13

**Authors:** Arantza Infante, Blanca Gener, Miguel Vázquez, Nerea Olivares, Arantza Arrieta, Gema Grau, Isabel Llano, Luis Madero, Ana Maria Bueno, Belén Sagastizabal, Daniela Gerovska, Marcos J Araúzo‐Bravo, Itziar Astigarraga, Clara I. Rodríguez

**Affiliations:** ^1^ Stem Cells and Cell Therapy Laboratory Biocruces Bizkaia Health Research Institute Cruces University Hospital Barakaldo Spain; ^2^ Service of Genetics Cruces University Hospital Barakaldo Spain; ^3^ Department of Pediatrics Biocruces Bizkaia Health Research Institute Cruces University Hospital Barakaldo Spain; ^4^ Department of Biochemistry, Immunology Unit Cruces University Hospital Barakaldo Spain; ^5^ Department of Pediatric Hematology, Oncology and Stem Cells Niño Jesús University Children´s Hospital Madrid Spain; ^6^ Department of Orthopedic Surgery Getafe University Hospital Madrid Spain; ^7^ Department of Endocrinology Getafe University Hospital Madrid Spain; ^8^ Computational Biology and Systems Biomedicine Research Group Biodonostia Health Research Institute Donostia Spain; ^9^ Department of Pediatrics Basque Country University UPV/EHU Leioa Spain

**Keywords:** cell therapy, mesenchymal stem cell, paracrine mechanism of action, regenerative medicine

## Abstract

**Background:**

Osteogenesis imperfecta (OI) is a rare genetic disease characterized by bone fragility, with a wide range in the severity of clinical manifestations. The majority of cases are due to mutations in the *COL1A1* or *COL1A2* genes, which encode type I collagen. Mesenchymal stem cells (MSCs), as the progenitors of the osteoblasts, the main type I collagen secreting cell type in the bone, have been proposed and tested as an innovative therapy for OI with promising but transient outcomes.

**Methods:**

To overcome the short‐term effect of MSCs therapy, we performed a phase I clinical trial based on reiterative infusions of histocompatible MSCs, administered in a 2.5‐year period, in two pediatric patients affected by severe and moderate OI. The aim of this study was to assess the safety and effectiveness of this cell therapy in nonimmunosuppressed OI patients. The host response to MSCs was studied by analyzing the sera from OI patients, collected before, during, and after the cell therapy.

**Results:**

We first demonstrated that the sequential administration of MSCs was safe and improved the bone parameters and quality of life of OI patients along the cell treatment plus 2‐year follow‐up period. Moreover, the study of the mechanism of action indicated that MSCs therapy elicited a pro‐osteogenic paracrine response in patients, especially noticeable in the patient affected by severe OI.

**Conclusions:**

Our results demonstrate the feasibility and potential of reiterative MSCs infusion for two pediatric OI and highlight the paracrine response shown by patients as a consequence of MSCs treatment.

## Graphical abstract

TERCELOI clinical trial addresses fundamental issues concerning the feasibility, security and potential of 5 MSCs infusions in two non immunosuppressed Osteogenesis Imperfecta pediatric patients. This trial allowed us to elucidate the mechanism of action of MSCs therapy, characterized by a pro‐osteogenic paracrine response. For that, we studied the protein and miRNA levels in sera from patients (collected before, along and after the cell treatment) besides unraveling the transcriptomic alterations due to those sera presence, and demonstrating their functional capabilities.

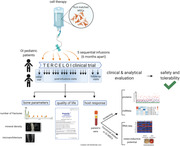

## 1 BACKGROUND

Osteogenesis imperfecta (OI), with an estimated incidence of 1 in 10 000‐20 000 births, is a rare connective tissue disorder affecting mainly the bones, which are extremely fragile due to low bone mass.^1^ OI is a highly clinically and genetically heterogeneous disorder.^2^ To date, mutations in 19 different genes have been described to cause OI with a wide range of clinical severity from mild to severe/lethal OI.^3^, ^4^, ^5^ The majority of cases (up to 90% of individuals) are caused by dominant mutations in the *COL1A1* or *COL1A2* genes.^6^ These genes code for collagen type I, the main extracellular matrix protein of bone that is composed of two α1 chains and one α2 chain to form a triple helix molecule.^7^ The severity of OI patients with *COL1A1* or *COL1A2* as causative genes depends on the nature of type I collagen mutations.^8^, ^9^, ^10^, ^11^, ^12^ Thus, mutations that lead to quantitative collagen defects result in structurally normal type I collagen at decreased levels, and are associated with a mild clinical phenotype. On the contrary, mutations leading to structural defects of type I collagen disrupt the folding of the triple helix and are associated with moderate‐severe‐lethal phenotypes. The remaining OI cases (∼10%), with moderate‐to‐severe clinical phenotypes, are originated by mutations in noncollagenous genes that encode proteins with crucial roles in collagen folding and posttranslational modifications, osteoblasts differentiation, and bone matrix mineralization.^1^ Moderate and severe OI patients suffer multiple low‐trauma fractures throughout their lifetime along with short height, skeletal deformities, and chronic pain. Because type I collagen is expressed in many tissues, extraskeletal manifestations are also common in these patients.^13^

Currently there is no cure for OI and the existing options are aimed at improving symptoms. Orthopedic surgery has been postulated as fundamental not only in the treatment of fractures, to strengthen the bone with mechanical measures, but to diminish the bone deformities associated with the disease.^13^ In the case of pediatric patients, pharmacological interventions such as antiresorptive drugs (mainly bisphosphonates) are focused on increasing bone mineral density (BMD) and decreasing pain.^13^, ^14^ This shortage of treatment options led Horwitz and coworkers to address a new therapeutic strategy based on mesenchymal stem cells (MSCs) transplantation into OI pediatric patients who previously were immunosuppressed.^15^, ^16^ The rationale for this approach was that after transplantation, the MSCs as the progenitors of osteoblasts would engraft in host tissues, differentiate into functional osteoblasts, and secrete normal collagen type I. This would ameliorate the symptoms associated with OI, as previously demonstrated in preclinical studies.^17^ Moreover, the reported cases of asymptomatic mosaic carriers of OI mutations supported this assumption, suggesting that having low amounts of cells with normal collagen would be enough to rescue or at least ameliorate OI pathological phenotypes.^18^ Notably, after cell therapy OI pediatric patients showed improvements in terms of growth velocity and fracture frequency, but these beneficial effects were transitory and the expected cell engraftment into bone tissue was anecdotal. Later studies addressing prenatal transplantation of fetal MSCs to OI patients also reported similar outcomes.^19^, ^20^ Thus, both prenatal and postnatal administration of MSCs in OI (one or two MSCs infusions) have been demonstrated to be feasible and safe, exerting clinical improvements of OI phenotypes, in spite of being short‐lived.^15^, ^16^, ^19^ The detailed mechanisms underlying the beneficial effects of MSCs therapy in OI are still unclear but the possibility of a paracrine therapeutic effect of MSCs is gaining attention.^21^, ^22^ However, there is no in vivo study exploring the trophic effects induced in OI patients by MSCs therapy.

Given the transitory effect of MSCs therapy in OI pediatric patients, we considered multiple histocompatible MSCs infusions with same‐donor cells as a strategy to improve cell therapy outcomes in OI patients. Thus, we conducted an independent, multicenter cell therapy phase I clinical trial based on reiterative infusions of nonmutated HLA‐identical or histocompatible (at least five shared out of six HLA antigens) allogenic MSCs applied to two OI pediatric patients. The principal aim of this trial was to assess the safety and secondarily the effectiveness of five (5‐6 months apart) MSCs infusions in OI patients irrespectively of their treatment with bisphosphonates and without subjecting them to any immunosuppressor treatment. At the same time, this study also sought to gain insight into the underlying paracrine mechanism induced by infused MSCs in an OI scenario. Importantly, the present clinical trial is the first, to the best of our knowledge, to explore not only the effects of serial MSCs infusions but also the mechanism of action of MSCs therapy in OI patients.

## 2 MATERIALS AND METHODS

### 2.1 Study design and patients

**FIGURE 1:**
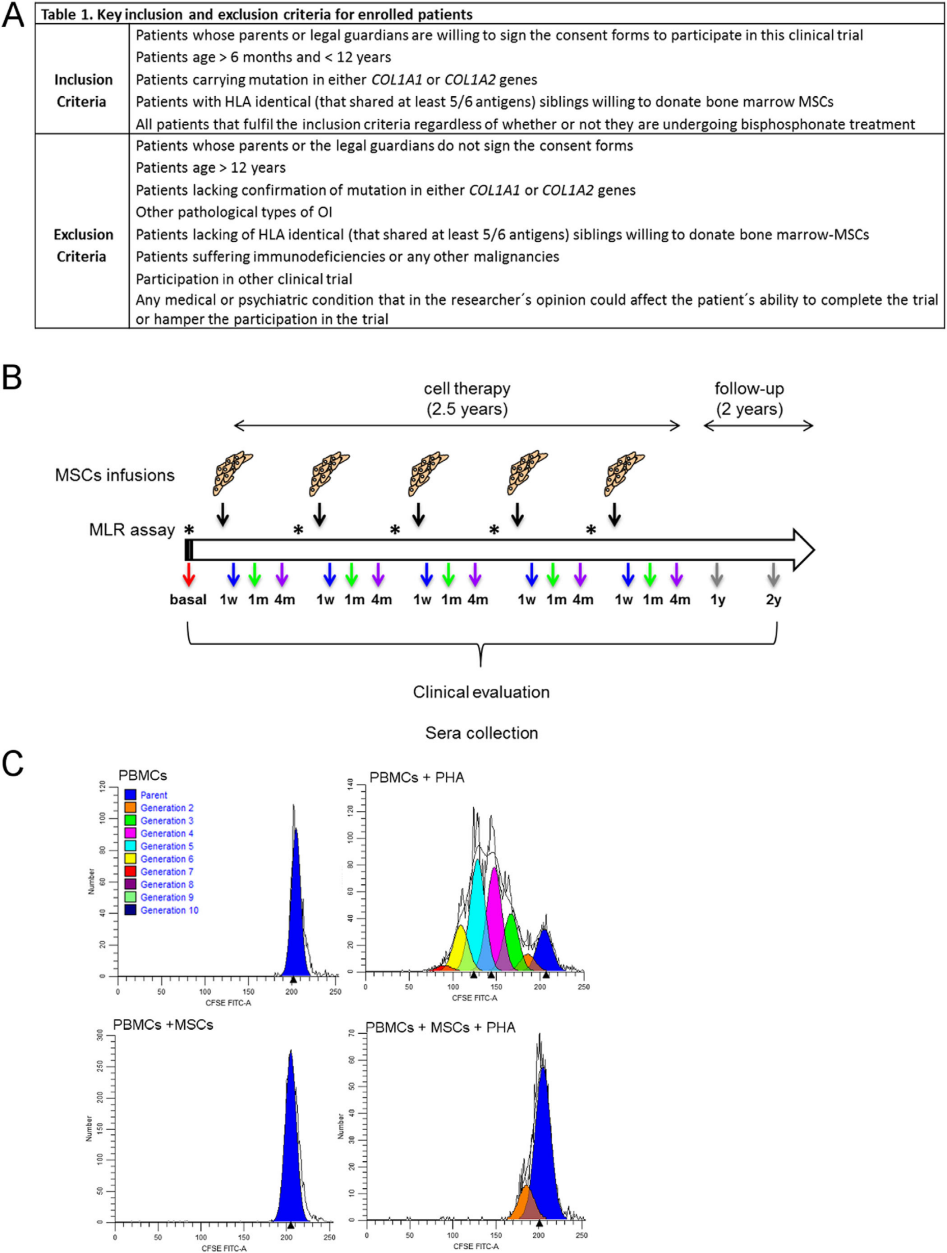
Overview of the TERCELOI clinical trial. (A) Table 1, reflecting the inclusion and exclusion criteria of the clinical trial. (B) Diagram illustrating the clinical trial workflow. The five MSCs infusions administered in total period of time of 2.5 years are indicated (black arrows). Previously to each cell infusion, mixed lymphocyte reaction assay was performed, denoted by asterisks. The visits for clinical and analytical evaluation in addition to sera collection after each cell infusion are denoted by colored arrows. Before the cell therapy: red. During the cell therapy: blue, 1 week (1 w); green, 1 month (1 m); and purple, 4 months (4 m) after each cell infusion. After the cell therapy: follow‐up visits at 1 and 2 years after the fifth cell infusion: gray. (C) Representative histogram plots of generations of divided cells as determined by ModFit‐LT software before the first MSCs infusion in P01 patient. Upper plots: controls consisted of unstimulated (left) or PHA‐stimulated PBMCs (right) isolated from P01. Dark blue peak in each plot indicates undivided parental cells. PHA‐stimulated PBMCs show several cell generations, denoting cell proliferation. Lower plots: P01 PBMCs cultured in the presence of donor MSCs, showing either no proliferation (left) or suppressed proliferation under PHA stimulation (right)

The Mesenchymal Stem Cell Therapy for the Treatment of Osteogenesis Imperfecta (TERCELOI) study was registered with clinicaltrials.gov (NCT02172885) and eudract.ema.europa.eu (2012‐002553‐38). TERCELOI is an independent multicenter phase I clinical trial to evaluate the feasibility, safety, and potential efficacy of infused sibling HLA‐matched MSCs in nonimmunosuppressed children with OI. Inclusion and exclusion criteria are specified in table 1 appearing in Figure 1A. Enrolled patients were the following: P01, male, 6 years 1 month of age and P02, female, 8 years and 1 month of age.

P01 was born to a healthy and nonconsanguineous Spanish couple. Skeletal dysplasia was suspected at 24 weeks of gestation. Cesarean delivery was at 34 weeks. At birth weight: 1770 g (p9, –1.4 SD), length: 39 cm (p < 1, –3.38 SD), and occipital frontal circumference (OFC): 30 cm (p3, –1.96 SD). X‐ray survey showed multiple fractures involving humeri, femur, tibia, and several ribs. A genetic study confirmed the clinical diagnosis of OI. A de novo heterozygous missense mutation was identified in exon 16 of *COL1A1* gene: c.1031G>A (p.Gly344Asp). The patient showed blue sclera and developed dentinogenesis imperfecta. He had normal neurodevelopment, sitting unsupported at the age of 9 months. He could never achieve unaided standing up or walking due to compression fractures of the spine and limb deformity. The first orthopedic surgical intervention was performed at the age of 4.5 years and consisted of correction of bilateral tibia angulation. Compassionate intravenous treatment with biphosphonates was initiated at the age of 57 days. He was treated with pamidronate until the age of 5 years and 4 months when zoledronate was prescribed instead. Spontaneous fractures or with minimal trauma and chronic bone pain have been common manifestations due to the extreme bone fragility. P02 was the first child born to a healthy and nonconsanguineous couple. Skeletal dysplasia was suspected at 34 weeks of gestation. Cesarean breech delivery was at 38 weeks of gestation. Weight:  2820 g (p 33; –0.44 SD) and OFC: 33 cm (p28; –0.4 SD). OI was suspected at birth. She had blue sclera, micrognathia, short lower limbs, and fracture of one clavicle. X‐ray skeletal assessment showed low bone density and prenatal fracture of the contralateral clavicle, eighth rib, and unilateral cubitus. Genetic studies identified a de novo heterozygous variant in the *COL1A2* gene: c.2133+6T>A resulting in the skipping of exon 35. At the age of 2 months, the patient revealed severe generalized osteopenia, and angulation of both femur and tibia with several areas of sclerosis. Compassionate treatment with pamidronate was initiated at the age of 15 months until the age of 7 years and 10 months when zoledronate was prescribed instead. She had normal social and language development. She could walk at the age of 3 years. Recurrent fractures of long bones of the upper and lower limbs leading to bone deformities required several surgical interventions. Mobility was largely wheelchair dependent.

### 2.2 HLA typing

Genomic DNA was extracted from both recipients and donors by peripheral blood using an automated method (MagNA re, Roche, USA). A ready‐to‐use system of HLA typing with PCR‐SSO methodology and Luminex flow analyzer technology was used to identify specific alleles present in a locus‐specific PCR‐amplified sample (LIFECODES, Immucor, USA) at the HLA‐A*, HLA‐B*, and DRB1* loci. PCR reactions were carried out in a Veriti thermal cycler (Applied Biosystems, USA) according to the manufacturer's recommendations. The results of HLA typing of each patient and donor (respectively) were as follows:

P01/donor (6/6 match): HLA‐A*03:01/*03:01; HLA‐A*11:01/*11:01; HLA‐B*15:18/*15:18; HLA‐B*52:01/*52:01; HLA‐DRB1*15:01/*15:01; HLA‐DRB1*15:02/*15:02.
P02/donor (5/6 match): HLA‐A*02/*02; HLA‐A*03/*03; HLA‐B*51/*51; HLA‐B*51/*51; HLA‐DRB1*07/*07; HLA‐DRB1*08/*14.

### 2.3 Isolation, characterization, expansion, and infusions of sibling bone marrow MSCs

HLA‐haploidentical MSCs were isolated from bone marrow aspirates obtained from healthy siblings. The samples were obtained at the Onco‐Hematology Service of the Niño Jesus Hospital in Madrid. The samples were transported following approved protocols to the Cell Production Unit of the Foundation for Biomedical Research at the Gregorio Marañón Hospital (UPC‐FIBHGM). MSCs were obtained under GMP rules at the UPC‐FIBHGM, with GMP certificate ES/125I/18, following the protocols of 12–088 IMPD approved by the Spanish Agency for Medicines and Health Products (AEMPs). Once the BM aspirates were received by the UPC‐FIBHGM personnel and their integrity was verified, the mononuclear cells (MNCs) were obtained using a density gradient (Ficoll Paque Premium, GE Healthcare, USA). The interface containing MNCs was washed twice and osmotic shock was performed to remove the remains of the Ficoll reagent and red cells, respectively. Then the MNCs were cultured at a concentration of 160 × 10^5^ cells/cm^2^ in complete DMEM medium (Gibco, USA), supplemented with 10% fetal bovine serum (FBS) (Gibco), and they were kept under standard culture conditions (37°C, 5% CO_2_, and 95% humidity). After 48 h, the appearance of the layer was reviewed and a medium change was carried out. This activity was performed every 3‐4 days until the layer achieved a confluence of 80%. At that point, the cells were harvested using Tryple Select following the guidelines of the manufacturer (Gibco). Cell counts and calculation of cell viability were carried out, and cells were subsequently seeded at a concentration of 5000 cells/cm^2^ to continue their expansion, repeating this process until obtaining the necessary cell number for the five doses applied to each patient, at a concentration of 4 × 10^6^ cells/kg of body weight. During the process, the following quality controls were carried out: cell count and viability, sterility test, mycoplasma detection, and differentiation potential (osteogenic, adipogenic, and chondrogenic differentiation). In addition, samples were taken to carry out the genetic stability analysis (CGH array) in order to have the result before releasing the doses. At the end of the culture, the cells were harvested, and the final product (FP) was prepared for infusion in the patients. For infusion, the cells were resuspended in Ringer Lactate (BRAUN, USA) supplemented with 1% human serum albumin. Cellular samples were taken from the FP for immunophenotypic characterization and the retention sample. The FP was packaged in a EVA bag (MacoPharma, USA) with a final volume of 20 mL. Patients were infused under noninvasive monitoring of vital signs. Each patient received five infusions of 4 × 10^6^ MSCs/kg each infusion, 5‐6 months apart.

### 2.4 Peripheral blood mononuclear cells isolation and serum collection

Venous blood samples were collected before the cell therapy (basal serum), during the cell therapy (1 week, 1 month, and 4 months after each MSCs infusion), and during the follow‐up period (1 and 2 years after the fifth and last MSCs infusion). The peripheral blood mononuclear cells (PBMCs) from TERCELOI patients were isolated by Ficoll density gradient‐based centrifugation (Lymphoprep, Axis‐Shield, Norway) following the manufacturer specifications at the specific time points indicated in the study design. The serum samples were collected in Clot Activator tubes (BD Vacutainer) and left undisturbed for 40 min at room temperature (RT) to allow the blood to clot. Then, blood samples were centrifuged at 1300 × *g* at RT during 15 min to remove the clot. The resulting serum supernatant was carefully transferred into a clean tube, aliquoted, and stored at –80°C until analysis.

### 2.5 Mixed lymphocyte reaction assay

PBMCs isolated from patients were resuspended in phosphate buffered saline (PBS) + FBS (5%) and stained with carboxyfluorescein succinate ester (CFSE) (Molecular Probes, USA) at a final concentration of 5 μM for 5 min, at RT in the dark. CFSE‐stained PBMCs were then washed three times with PBS + FBS (5%). To perform the mixed lymphocyte reaction (MLR) assay, triplicate samples of the CFSE‐stained PBMCs were cultured in a 96‐well plate (200 000 cells/well), where the MSCs from HLA‐matched siblings were seeded the day before (20 000 cells/well). The cells were cultured in complete RPMI medium (Invitrogen, USA) for 5 days with or without the presence of phytohemagglutinin (PHA). Cultures of unstimulated (negative control) and PHA (Sigma‐Aldrich, USA)‐stimulated PBMCs (2 μg/mL) were seeded without the presence of MSCs. Each condition was seeded in triplicate. After 5 days, the OI PBMCs were harvested, stained with antihuman‐CD3 antibody (Becton Dickinson, USA), and acquired by flow cytometry. Cell proliferation was calculated using ModFit LT (Verity Software House, USA). The parental generation was set with unstimulated PBMCs, which show a unique peak of fluorescence. Then, cell division was tracked by measuring dilution of CFSE signal in stimulated cells.

### 2.6 Bone mineral density

BMD at the lumbar spine, from L1 to L4, was measured by dual energy X‐ray absorptiometry on a whole‐body scanner in a pediatric platform (Hologic QDR densitometer).

### 2.7 Assessment of bone microstructure

X‐ray images from the femoral distal metaphysis of patients were used to manually delineate a region of interest (ROI). This ROI was drawn by selecting only the region where trabecular bone was present, that is, avoiding cortical bone, metallic implants, and fracture callus. Once the ROI was delineated, signal heterogeneities were corrected and through an Otsu's thresholding algorithm, the ROI was binarized separating trabecular bone (foreground) from marrow (background). To characterize bone microstructure, different morphological imaging biomarkers (explained in Methods in the Supporting Information) were automatically calculated from the ROI by using Quibim Precision Software (QUIBIM, Valencia, Spain).^23^, ^24^, ^25^ Bone volume to total volume (BV/TV) ratio is the percentage of bone within the ROI. The number of voxels corresponding to trabecular bone were divided by the number of all the voxels (trabecular bone and marrow). Mean trabecular bone thickness (TbTh) is the mean thickness value of all the beams of trabecular bone within the ROI. To calculate the trabecular thickness on each pixel, a pipeline of different morphological and level‐based image processing techniques was applied to the binary image. Mean trabecular bone separation (TbSp) is the mean size of the intertrabecular spaces or pores. Trabecular number (TbN) is the ratio between the percentage of bone and the mean trabecular thickness. Quality Trabecular Score (QTS) score was obtained from a multivariate analysis based on principal component analysis (PCA) over a population with previously defined imaging biomarkers. This score assesses trabecular bone quality, with lower values indicating a higher fracture risk.

### 2.8 Assessment of health‐related quality of life

Health‐related quality of life (HRQoL) was assessed by PedsQL version 4.0, which is a validated modular approach to measuring HRQoL in healthy children and adolescents (ages, 2‐18) and those with acute and chronic health conditions.^26^ The application of PedsQL has also been recommended by the Initiative on Methods, Measurement and Pain Assessment in Clinical Trials (IMMPACT).^27^ PedsQoL 4.0 consists of 23 questions measuring the core health dimensions delineated by World Health Organization. Thus, it encompasses four domains: physical (eight items), emotional (five items), social (five items), and school (five items) functioning. We applied the questionnaire to patients (self‐report) and parents (proxy‐report). Importantly, the items for self‐report and proxy‐report are basically the same differing in developmentally appropriate language. The questions refer to the frequency of problems that occurred during the past month. A 5‐point response scale is used to measure the items (0 = never a problem, 1 = almost never a problem, 2 = sometimes a problem, 3 = often a problem, and 4 = almost always a problem). Then, items are reverse scored and lineally transformed to a 0‐100 scale (0 = 100, 1 = 75, 2 = 50, 3 = 25, and 4 = 0) meaning that higher scores indicate a better level of HRQoL.

### 2.9 Human glycosylphosphatidylinositol‐specific phospholipase D1 detection

Sera from P01 and P02 collected before, during (1 week, 1 month, and 4 months after each MSCs infusion), and after the cell therapy were used for human glycosylphosphatidylinositol‐specific phospholipase D1 (GPLD1) detection. For this, human GPLD1 enzyme‐linked immunosorbent assay (ELISA) kit (FineTest, China) was used with diluted sera samples (1:400), used in duplicates, following manufacturer's instructions.

### 2.10 Antibody array assay for serum profiling

For antibody array experiments, sera from P01 and P02 collected before, 1 week, 1 month, and 4 months after the first MSCs infusion were used. The relative expression of more than 1000 human serum proteins was performed by using the RayBio Label‐based (L‐Series) Human Antibody Array 1000 kit (AAH‐BLG‐1000) according to the manufacturer's instructions (RayBiotech, USA). Scanned images were captured using an Axon GenePix laser scanner and analyzed using RayBio Analysis Tool software. Protein intensities were obtained by taking the average of the two spots specific to each protein, and all protein intensities on a given array were normalized against the blank, negative control, and positive control spots.

### 2.11 MicroRNAs expression analysis

The expression of 21 circulating microRNAs (miRNAs) related to bone quality and musculoskeletal diseases were analyzed by using the OsteomiR test (TamiRNA GmbH, Austria). Sera samples from TERCELOI patients before, during (1 month after each cell infusion), and after the cell therapy were used as starting materials to extract miRNAs. Then, cDNA synthesis and Q‐PCR were performed. Importantly, for each step, specific controls were used: unisp4 for the extraction step, nonhuman cel‐miR‐39 for the cDNA synthesis, and Unisp3 IPC as an interplate calibrator. The existence of hemolysis in the serum samples was excluded by studying the difference between has‐miR‐451a and has‐miR‐23‐3p. For normalization of expression data, an RNAspike‐in control was used. Raw data were converted into Cq‐values according to the second derivative method and then transferred to the ClustVis online web tool for plotting the expression values as a heatmap.

### 2.12 OI MSCs isolation and characterization and culture

OI MSCs were derived from discarded and donated bone fragments of OI pediatric patients (carrying mutations in either *COL1A1* or *COL1A2*) undergoing corrective surgery. The donation was approved by the Basque Clinical Research Ethics Committee. Briefly, bone chips were mechanically flushed with PBS and then cut into small pieces to extrude cells without the use of enzymes. The bone pieces were left undisturbed during 14 days until OI MSCs migrated to cell culture plate. OI MSCs were assessed for the expression of CD105, CD90, and CD73 and the absence of CD14, CD34, CD45, CD19, and HLA‐DR and were also tested for their potential to differentiate to osteoblasts and adipocytes using specific cell culture media.

OI MSCs were cultured under standard growth medium (DMEM low glucose) with glutamax (Gibco, USA), penicillin/streptomycin (Gibco), and fetal bovine serum at 10% (Sigma‐Aldrich, USA). Osteogenesis induction medium (OIM) was composed of standard medium plus ascorbic acid 0.2 mM, β‐glycerophosphate 10 mM, and dexamethasone 10 nM (all from Sigma‐Aldrich). When specified, FBS was replaced by P01 and P02 serum samples at 2.5%.

### 2.13 RNAseq and Q‐PCR validation

OI MSCs from 10 pediatric patients were seeded in 96‐well plates (1000 cells/well) and the following day cultured in the presence of OIM. After 4 days of differentiation, total RNA was isolated from cells with the AllPrep DNA,RNA,MicroRNA extraction kit (QIAGEN, USA). cDNA library (TrueSeq stranded Total cDNA library, Illumina, USA) and sequencing at HiSeq 4000 (PE100nt, 50 million reads/sample) was performed by Macrogen (South Korea). HISAT2 was used to align the RNA‐seq reads for each of the 20 samples to the human reference genome GRCh38.^28^ Cufflinks was used to assemble the mapped reads into possible transcripts and generate a transcriptome assembly.^29^ The counts of aligned reads to each gene were calculated with HTSeq.^30^ We used in‐house software to merge the expression results into a single text file used in the downstream analysis in Matlab (MathWorks). We performed quantile normalization to equalize the data and stabilized them through the log_2_ transform of the data plus one. The heatmap of the most highly variable transcripts, the hierarchical clustering dendrograms (calculated using the unweighted pair group method with arithmetic mean and Euclidean distance measure), and the PCAs were performed using in‐home functions developed in Matlab (MathWorks). We designed a democratic method to select differentially expressed genes (DEGs) between the two conditions: after the cell therapy versus before the cell therapy. We searched for upregulated genes after treatment by giving each gene a “for” vote in case of upregulation of more than a threshold *θ*_vote_ in log_2_ scale in the after treatment condition compared to the before treatment condition, and an “against” vote in case of downregulation of more than *θ*_vote_ in log_2_ scale. We searched for downregulated genes after treatment by giving each gene a “for” vote in case there was a downregulation of more than *θ*_vote_ in log_2_ scale in the after treatment condition compared to the before treatment condition, and an “against” vote in case of upregulation of more than *θ*_vote_ in log_2_ scale. Finally, we chose all the genes with nine or more “for” votes for both up‐ and downregulation. We used the *P*‐value produced by a tailed paired *t*‐test to rank the selected genes. We chose a threshold *θ*_vote_ = 0.1. To assay gene‐coverage and obtain track plots, we sorted the alignment bam files with samtools and produced the bed files with the bedtools.^31^, ^32^ We developed a function in Matlab (MathWorks) that for each gene of interest takes the exon boundary information from the basic annotation file in gtf format from gencode (https://www.gencodegenes.org/human/) version 33, and plots the gene‐coverage count track plots, keeping for the tracks of the same gene in all the samples the same scale. The RNAseq data have been deposited in Gene Expression Omnibus.

The same RNA samples used for RNAseq were used for validation by Q‐PCR. For this, 1 μg of the same RNA used for RNAseq was reverse transcribed using the Superscript III First Strand cDNA kit (Thermo Fisher Scientific) according to the manufacturer's instructions. For each gene, all reactions were carried out in triplicate on an AriaMX Real Time PCR System (Agilent, USA) using Brilliant III Ultrafast SYBR Green Master mix (Agilent). The program used consisted of denaturation of the samples at 95°C for 2 min, annealing step at 60°C for 30 s, and extension step at 72°C for 30 s. For gene expression normalization, *GAPDH* was used. The gene expression levels were calculated using the Δ*CT* method and are shown as the mean value with the standard error of the mean. Primer sequences are available upon request.

### 2.14 Tissue nonspecific alkaline phosphatase activity measurement

OI MSCs from eight pediatric patients were seeded in quadruplicates in 96‐well plates (1000 cells/well) and the following day induced to differentiate in the presence of OIM and serum (2.5%) from TERCELOI patients. After 4 days of differentiation, tissue nonspecific alkaline phosphatase (TNAP) activity was determined by adding to each well a chromogenic substrate of alkaline phosphatase, 1‐step PNPP substrate solution (Thermo Fisher Scientific, USA) and absorbance was measured at 405 nm. To normalize TNAP activity, cell number was determined by Cell Counting Kit‐8 (CCK‐8, Bimake, USA) and absorbance at 415 nm was measured in a parallel plate. As negative controls, OI MSCs cultured under nondifferentiation conditions were used.

### 2.15 Alizarin red S staining

OI MSCs from two pediatric patients were seeded in triplicates in 12‐well plates (15 000 cells/well) and the following day induced to differentiate in the presence of OIM supplemented with serum samples obtained from TERCELOI patients at 2.5%. Medium was changed twice per week. After 14 days, mineralization was assessed by staining the extracellular calcium deposits. Cells were fixed with 4% paraformaldehyde (Sigma‐Aldrich) and stained with alizarin red S staining (Thermo Fisher Scientific) (2%) for 45 min. Then, to quantify the mineralization, the wells were washed twice and the dye was extracted with 10% cetylpyridinium chloride (Sigma‐Aldrich) in sodium phosphate buffer (Sigma‐Aldrich). Absorbance was measured at 560 nm in a plate reader.

### 2.16 Statistical analysis

For the Q‐PCR validation and TNAP activity measurements, the statistical analysis was conducted using GraphPad Prism (Version 7.0) software. We used Mann‐Whitney *U* test for comparing two groups. *P*‐values were represented by asterisks as follows: *P value < 0.05 and **P value < 0.01. The mean values and standard error of the mean were calculated.

## 3 RESULTS

### 3.1 Clinical trial design, safety, and tolerability

Two patients fulfilling the inclusion/exclusion criteria summarized in table 1 appearing in Figure 1A were enrolled, P01 (6‐year‐old boy), affected by severe OI, and P02 (8‐year‐old girl), affected by a moderate form of OI. The patients received five MSCs infusions, 5‐6 months apart, which spanned 2.5 years (Figure 1B). Clinical and analytical evaluation (biochemistry, nutritional status, immunology, hematology, and calcium phosphate metabolism), bone parameters (fracture rates, BMD, and microstructure), and HRQoL, besides sera samples, were analyzed before (basal), during (1 week, 1 month, and 4 months after each infusion), and after (follow‐up period: 1 and 2 years since the fifth and last MSCs infusion) the cell treatment (Figure 1B). Among the clinical parameters monitoring, immunological features were given thorough consideration; in addition to immunoglobulins (IgG, IgM, and IgA), anti‐HLA, and antinuclear antibodies, the monocytes and lymphocytes subsets (T, B, and NK) were analyzed finding no alteration. Moreover, to ensure an absence of host immune response to sibling MSCs, functional evaluations based on in vitro MLR assay were performed prior to each MSCs administration (Figure 1B). Donor HLA‐matched MSCs from siblings were co‐cultured with PBMCs isolated from patients without triggering the activation of the latter (Figure 1C). Additionally, co‐culture of donor allogenic MSCs with PBMCs stimulated with PHA resulted in an inhibition of the proliferation of PHA‐activated PBMCs (Figure 1C).

Of note, the five reiterative infusions of MSCs were well tolerated in both patients and there were no treatment‐related adverse events either during the cell therapy, nor during the follow‐up period.

### 3.2 Bone parameter outcomes

**FIGURE 2:**
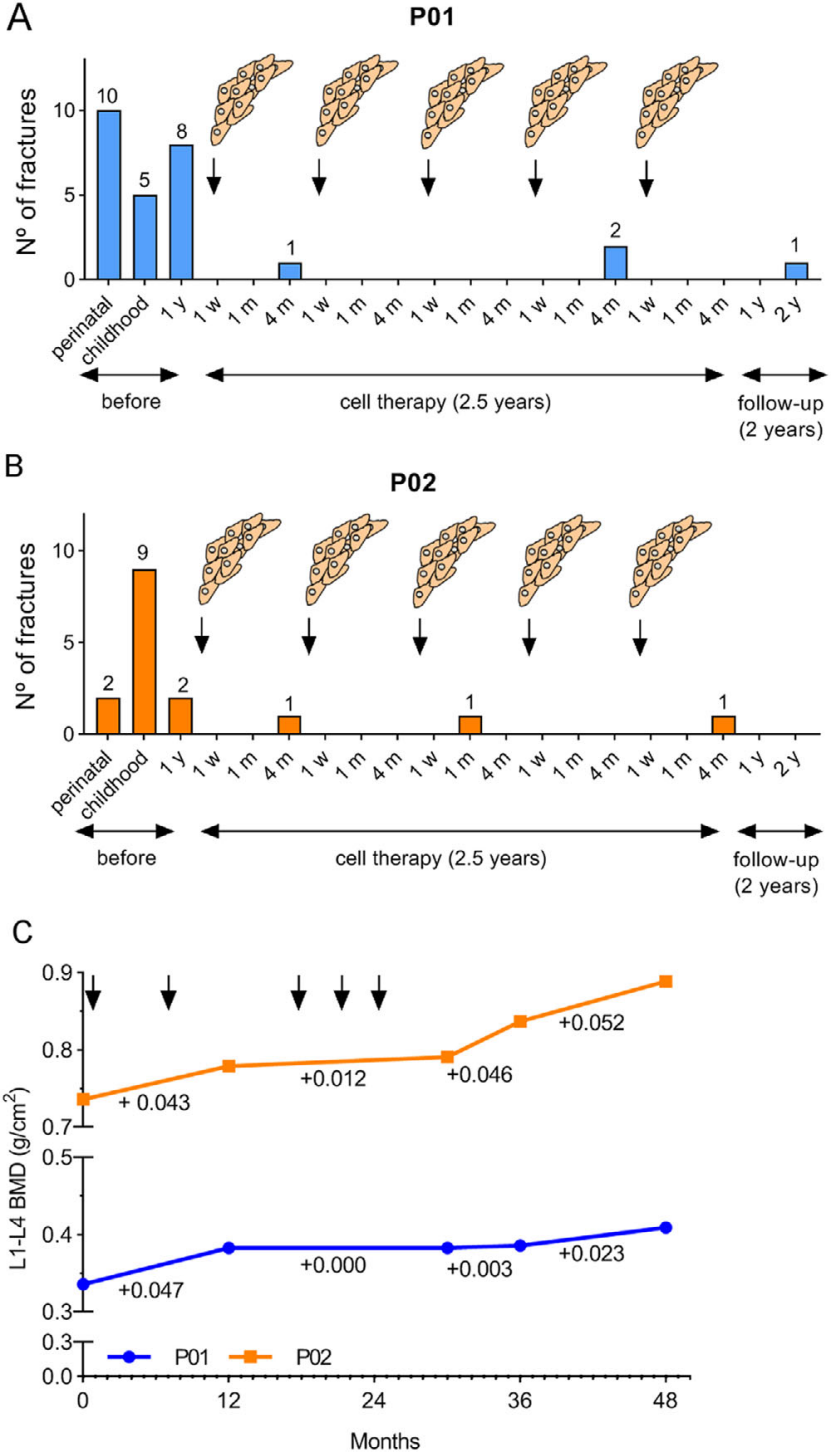
Bone‐related parameters measured in OI patients before, during, and after the MSCs therapy. (A and B) Number of fractures in TERCELOI patients before the cell therapy: during the perinatal period, childhood (from perinatal period up to 5‐year old in P01 and up to 7‐year old in P02), and the year previous the first infusion of MSCs; during the cell therapy treatment (each of the five MSCs infusion is depicted) and during the follow‐up visits (1 and 2 years after the fifth MSCs infusion); 1 w (1 week), 1 m (1 month), and 4 m (4 months) indicate the visits after each cell infusion. (C) BMD of P01 (blue line) and P02 (orange line) before (0 months), during (MSCs infusions are specified with arrows), and after the cell therapy

In accordance with their severe (P01) and moderate (P02) OI, both patients had a significant number of reported fractures from birth and through childhood: 15 in the case of P01 (Figure 2A) and 11 in the case of P02 (Figure 2B). During the year prior to MSCs therapy, P01 had eight more documented fractures, and P02 had two. Remarkably, during the 2.5 years of cell treatment, P01 showed a reduction in the number of fractures (three documented fractures), whereas P02 reported only two fractures. Moreover, this effect was maintained in both patients until the end of the follow‐up visits. In order to assess the effects of cell therapy in the bone tissue of TERCELOI patients, lumbar spine BMD was analyzed (Figure 2C). As expected, the basal measurements of BMD in patients before the cell therapy were in accordance with their OI severity. Thus, P01, the most severe TERCELOI patient (6‐year old), showed a BMD of 0.336 g/cm^2^, which corresponds to healthy 2‐3 years’ old children.^33^ In contrast, P02 exhibited higher basal BMD values: 0.736 g/cm^2^ that approximate the estimated BDM in normal healthy children of that age (8 years).^33^ Strikingly, both patients showed a substantial and similar BMD increase trend after the first two MSCs infusions (+0.043 and +0.047 for P01 and P02, respectively). P01's BMD remained steady after the first two infusions, until the final measurement (at the 2 years’ follow‐up visit), when he again showed an increase of 0.023 g/cm^2^. In the case of P02, she showed a gradual improvement of BMD during the duration of the cell therapy treatment, a trend that was especially noticeable at the follow‐up visits.

**FIGURE 3:**
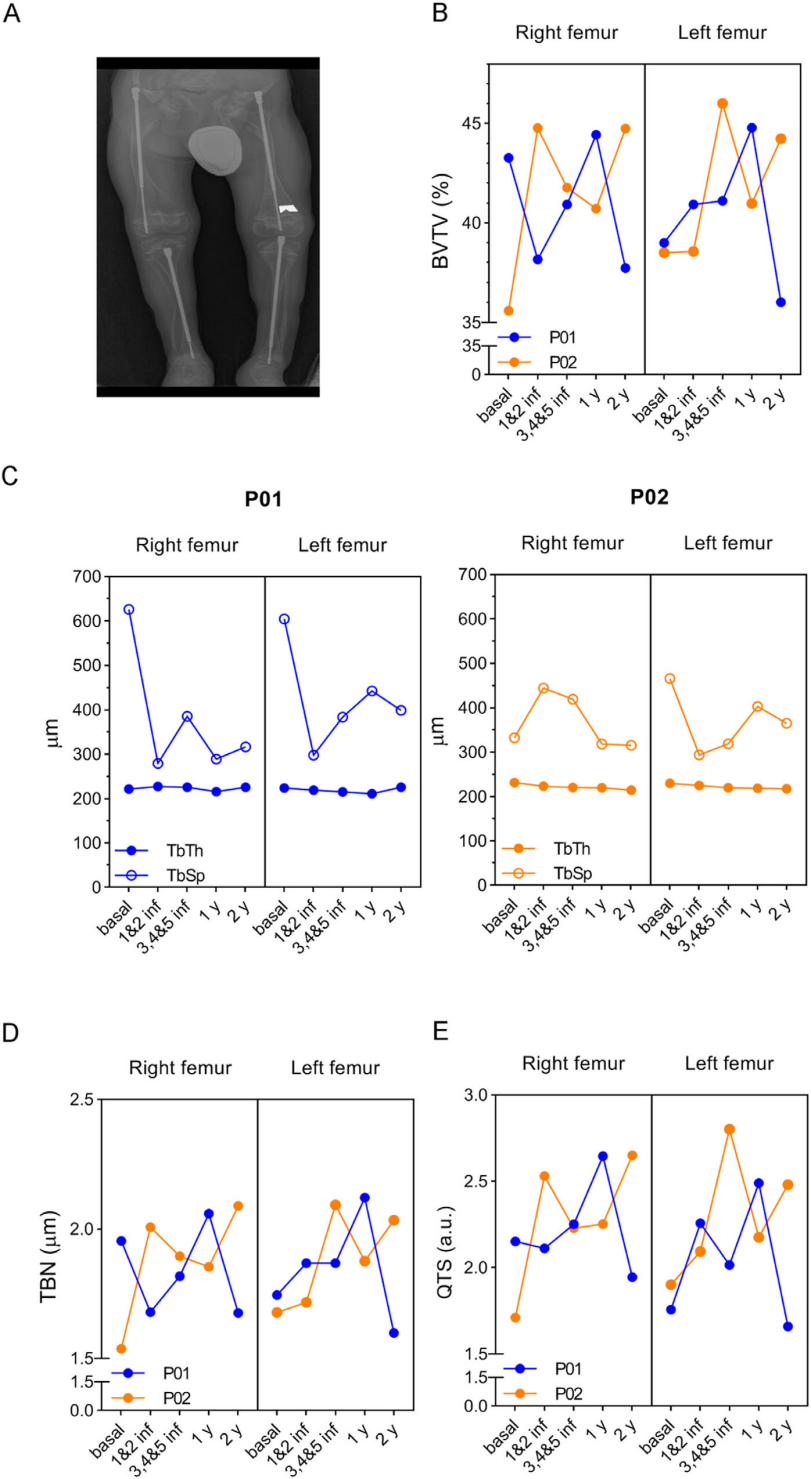
Trabecular bone microarchitecture from TERCELOI patients before, during, and after the cell therapy. (A) Representative image of QUIBIM software, indicating the ROI used to determine the different bone parameters in patients before (basal), after the two first infusions (1 and 2 inf), after the three last infusions (3, 4, and 5 inf), and during the follow‐up visits (1 and 2 y). (B) Percent (%) of bone (BVTV). (C) Trabecular thickness (TbTh) and separation (TbSp). (D) Trabecular index (TBN). (E) Quality of trabecular structure (QTS). P01, blue color lines; P02, orange color lines

We also analyzed the trabecular bone microstructure from right and left femoral distal metaphysis of TERCELOI patients, by using Quibim Precision Software, a quantitative imaging tool (Figure 3A). This analysis showed general improvements in the bone microstructure parameters. P02 showed an increase in the trabecular femoral bone volume (BV/TV) during the whole clinical trial period. P01 also showed an increase in BV/TV, which was especially noticeable after five MSCs infusions, although this value dropped in the last follow‐up visit (Figure 3B). The trabecular thickness (TbTh) remained constant throughout the clinical trial; however, the trabecular separation (TbSp), reflecting the mean size of trabecular pores in the bone, showed a drastic drop in P01, which was especially noticeable after the two first MSCs infusions (Figure 3C). In the case of P02, with a lower basal TbSp value, this parameter did not have a pronounced change. The trabecular number (TbN) also showed improvements in both patients, with a similar trend as that showed in BV/TV measurement: P02 showed a continuous increase during the whole clinical trial, whereas P01 showed an initial increase, which subsequently fell by the 2‐year follow‐up visit (Figure 3D). Finally, we studied the quality of trabecular structure (QTS) of patients by using the QTS score, an index that encompasses all the previous parameters analyzed. Both patients showed improvements in the QTS score after cell infusions (Figure 3E). Importantly, P02 finished the clinical trial with a higher QTS than that showed before the cell therapy. P01's QTS values, however, returned to pretreatment levels by the second follow‐up. These negative outcomes in the P01's second follow‐up visit indicated a deterioration of bone microstructure. This circumstance was consistent with the fact that in the 2‐3 months after the last follow‐up visit, P01 suffered three additional fractures.

Overall, these findings suggest that MSCs therapy had a beneficial effect in the bone tissue of patients, leading to a notable lower number of fractures, which was noticeable from the early stages of treatment. Moreover, the decline in some bone parameters in the second follow‐up visits with P01 suggests the necessity of continuous treatment, especially in the most severe patients.

### 3.3 Quality of life and achievements of functional activities

**FIGURE 4:**
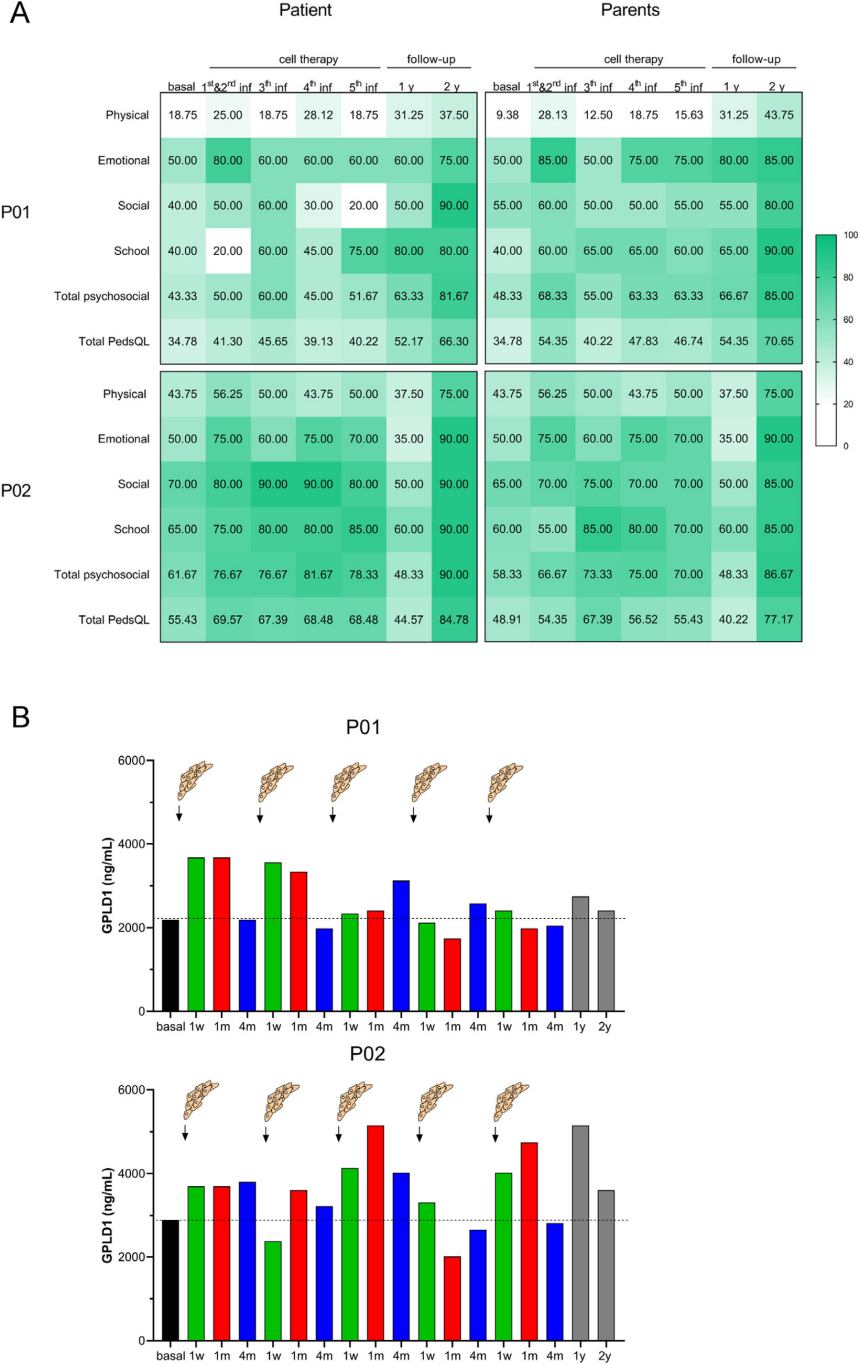
Quality of life and GPLD1 levels of patients during TERCELOI. (A) At that indicated time points: before (basal), during (after the first, second, third, fourth, and fifth infusions), and after the MSCs therapy (1 and 2‐year follow‐up visits) questionnaires were performed to each patient and his/her parents. The obtained numerical scores ranging from 0 to 100 have been depicted with a green color scale. (B) Serum GPLD1 detection by ELISA in P01 (upper graph) and P02 (lower graph) at the mentioned time points

Children with OI often struggle with chronic pain, even during periods without fractures.^34^ This has a considerably negative impact on quality of life, interfering with daily activities and leading to poor mobility, missed school days, and psychosocial stress. Thus, HRQoL measurement in OI is essential to evaluate the clinical outcomes, especially those regarding cell‐based therapies.^35^ To address this point and evaluate functional improvements in TERCELOI patients, we used the PedsQL questionnaire, which has been previously employed with OI pediatric patients and their parents. This validated survey consists of 23 items in four functioning areas: physical, emotional, social, and scholastic.^14^, ^36^, ^37^ The scores obtained from patients and parents were quite similar across all the time points assessed, denoting an internal consistency in the subjective perception of achieved improvements (Figure 4A). In accordance with his more severe phenotype, before, during, and after cell therapy, P01's HRQoL was generally more affected (lower values) than P02's. Overall, both patients showed improvements in all the parameters assayed from the beginning of cell therapy. Importantly, this effect was maintained during the entire clinical trial, with the exception of the 1‐year follow‐up visit in P02, possibly associated with entry into early adolescence and her coping with an acceptance of the illness. Psychological support helped to overcome the situation as reflected in the 2‐year follow‐up visit, which showed the highest scores in all parameters assayed for both patients. During the treatment, there were remarkable clinical findings that supported the improved outcomes of HRQoL of patients: for example, P01 (6‐year old) referred a significant perception of wellness and increase strength and mobility before the third infusion of MSCs. Among several improvements, it is worth mentioning that for the first time in life he was able to roll, from lying on his back, to a prone position and slide toward the edge of a sofa or bed and stand on his feet for a few seconds. Likewise, P02 (8‐year old) indicated that she felt confident enough and had the strength to try physical activities (eg, try a cartwheel) for the first time in her life after receiving the second infusion of MSCs.

Increased blood concentrations of GPLD1 have been found in active (exercised) aged mice and healthy elderly humans when compared to their sedentary counterparts.^38^ In fact, the administration of GPLD1 has been suggested as a therapeutic approach providing the benefits of exercise in old age.^38^ Interestingly, GPLD1 is also known to be expressed in the developing bone of mouse embryos.^39^ Taking these GPLD1 features into account, we tested whether cell therapy could modulate GPLD1 levels in TERCELOI patients, which showed improvements in the HRQoL and in bone parameters during the clinical trial. Strikingly, serum GPLD1 levels in P01 rose immediately (1 w and 1 m) after the two first infusions and then dropped to baseline levels 4 months later (4 m) (Figure 4B). In the case of P02, this effect after the two first infusions was not as remarkable as the one observed in P01, showing the highest GPLD1 levels 1 month after the third cell infusion.

### 3.4 Protein and miRNA screening in sera from patients

**FIGURE 5:**
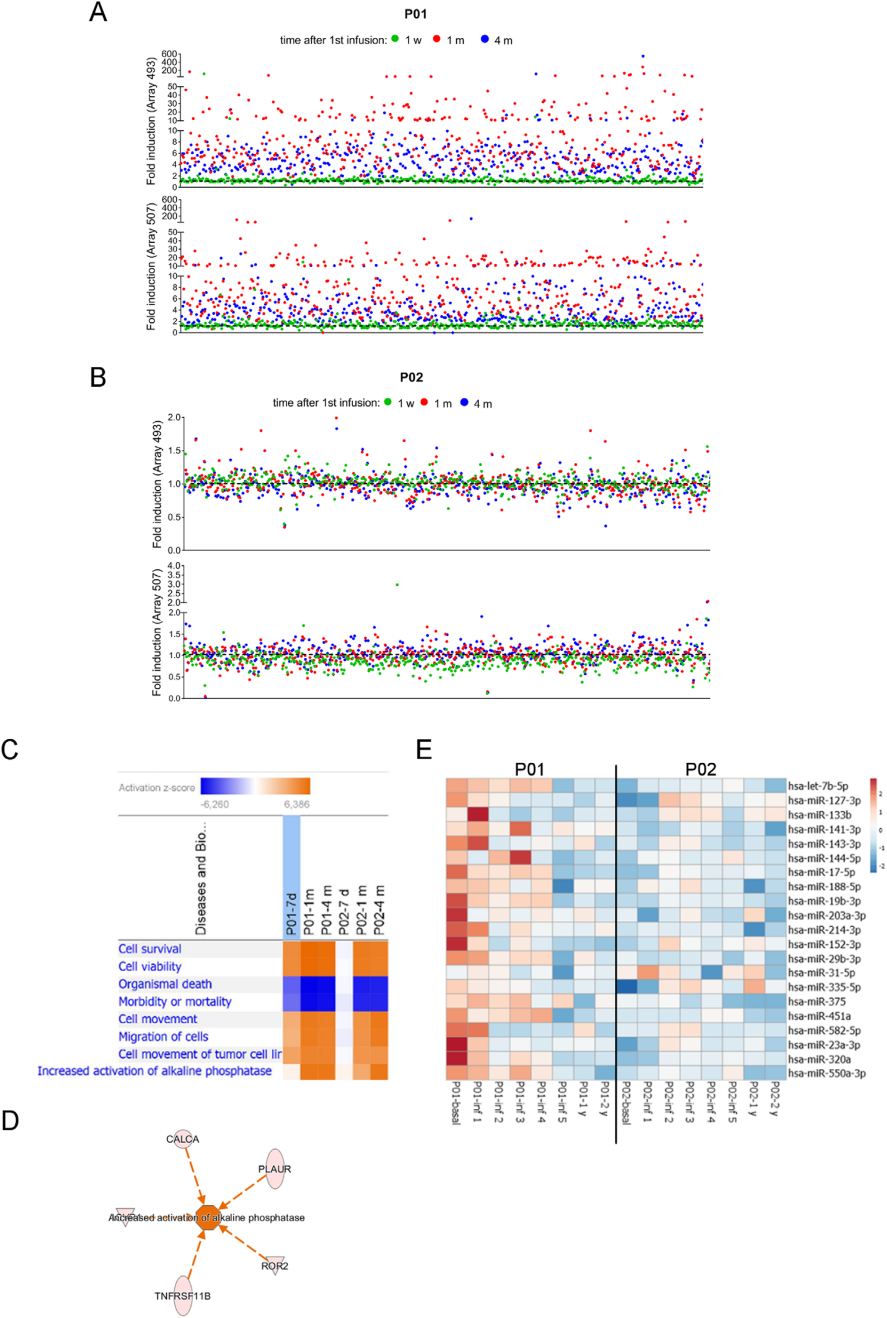
Expression analysis of proteins and miRNAs in sera of patients. (A and B) Antibody arrays quantification of serum proteins in TERCELOI patients. The dot plots reflect the expression fold induction of the 1000 proteins included in both arrays (493 and 507) after the first MSCs infusion (1 w, green color; 1 m, red color; 4 m, blue color) when compared to the expression of those proteins before the cell therapy (black, dashed line). Each dot represents a protein. (C) IPA comparison analysis for commonly upregulated proteins (fold change > 1.3) after the first MSCs infusion (1 w, 1 m, and 4 m) in P01 and P02. The heatmap represents the significantly enriched (*P* < .05) biological functions predicted to be activated (*z*‐score > 2) or inhibited (*z*‐score < 2). (D) Activation network diagram for TNAP predicted by IPA based on five proteins found to be upregulated in P01 and P02 1 month after the first cell infusion. (E) Heatmap of the miRNAs expression signature of P01 and P02 serum samples, before, during (1 month after each cell infusion), and after (1 and 2 years’ follow‐up) cell therapy. Unit variance scaling was applied to the global mean normalized expression values for each miRNA. Color indicates relative upregulation (red) or downregulation (blue) for each miRNA (row)

To gain further insight into the cellular and molecular mechanisms driven by cell therapy in TERCELOI patients, we explored the possibility of a paracrine response induced by infused MSCs. Sera from patients were obtained before, during, and after the cell therapy (Figure 1B). First, we addressed an unbiased simultaneous screening of serum protein expression. For this, we used a biotin label‐based antibody array technique, capable of detecting the expression of 1000 proteins covering a wide range of functions. To perform this analysis, for each patient, we compared the sera obtained after the first cell infusion collected at 1 week (1 w), 1 month (1 m), and 4 months (4 m) versus the basal serum (collected before the cell therapy). Strikingly, 1 w and 4 m sera from P01 showed a great increase in the expression levels of almost all the proteins included in the antibody array when compared to his basal serum levels (Figure 5A). Noticeably, this effect was very pronounced in a subset of proteins from 1 m serum, which exhibited a more than ninefold difference. When analyzed with the Database for Annotation, Visualization and Integrated Discovery (DAVID) software, these proteins could be grouped into enriched categories related to inflammatory response, angiogenesis, chemotaxis, collagen catabolic process, and BMP, TGF‐β, and JAK/STAT signaling pathways (Table S1). In the case of P02, the modulation of sera protein expression by MSCs therapy was not as obvious, and the increase in fold change of differentially expressed proteins was modest (Figure 5B). We found that more than 100 proteins were commonly upregulated in sera from TERCELOI patients after the cell therapy. QIAGEN Ingenuity Pathway Analysis (IPA) showed that these upregulated proteins were grouped in enriched functional categories related to “cell migration” and “cell survival,” which were predicted to be activated (positive *z*‐score) (Figure 5C). Moreover, a subset of proteins in the category “increased alkaline phosphatase expression” was discovered, especially in the 1 m and 4 m sera (Figures 5C and 5D). These findings suggest that the MSCs infusion can induce a deep host systemic response, especially in the most severe patient.

miRNAs are noncoding 22 nucleotide RNAs that repress gene expression at the posttranscriptional level and can play important roles in some diseases.^40^ In fact, specific circulating miRNA profiles have been described to be associated with increased risk of fragility fractures.^41^, ^42^ Therefore, we decided to analyze the expression of fracture‐associated miRNAs in the sera of patients collected before, during (1 month after each cell infusion), and after the MSCs therapy (follow‐up visits). In our study, 21 miRNAs known to be associated with high‐risk of fragility fractures were screened in P01 and P02 patients. Before cell therapy, P01 basal serum showed a generally high expression of the assessed miRNAs (Figure 5E). Strikingly, the expression of the great majority of these miRNAs displayed a continuous and progressive downregulation during the cell therapy, and through the follow‐up visits. Of note, the higher downregulation of miRNAs expression was achieved after the fifth MSCs infusion. In the case of P02, the miRNAs’ expression pattern before cell therapy was in general terms the opposite as that shown by P01, and thus the majority of the miRNAs were slightly downregulated (Figure 5E). Moreover, the cell therapy did not show a noticeable change in miRNAs expression, because miRNAs’ downregulation remained in the follow‐up visits. Even so, there were some miRNAs, known to play specific roles as osteogenesis inhibitors, whose expression in the follow‐up visits were even lower than before the cell therapy: miR188‐5p, miR214‐3p, and miR375.^43^, ^44^, ^45^

### 3.5 Functional analysis of sera from patients in OI MSCs

**FIGURE 6:**
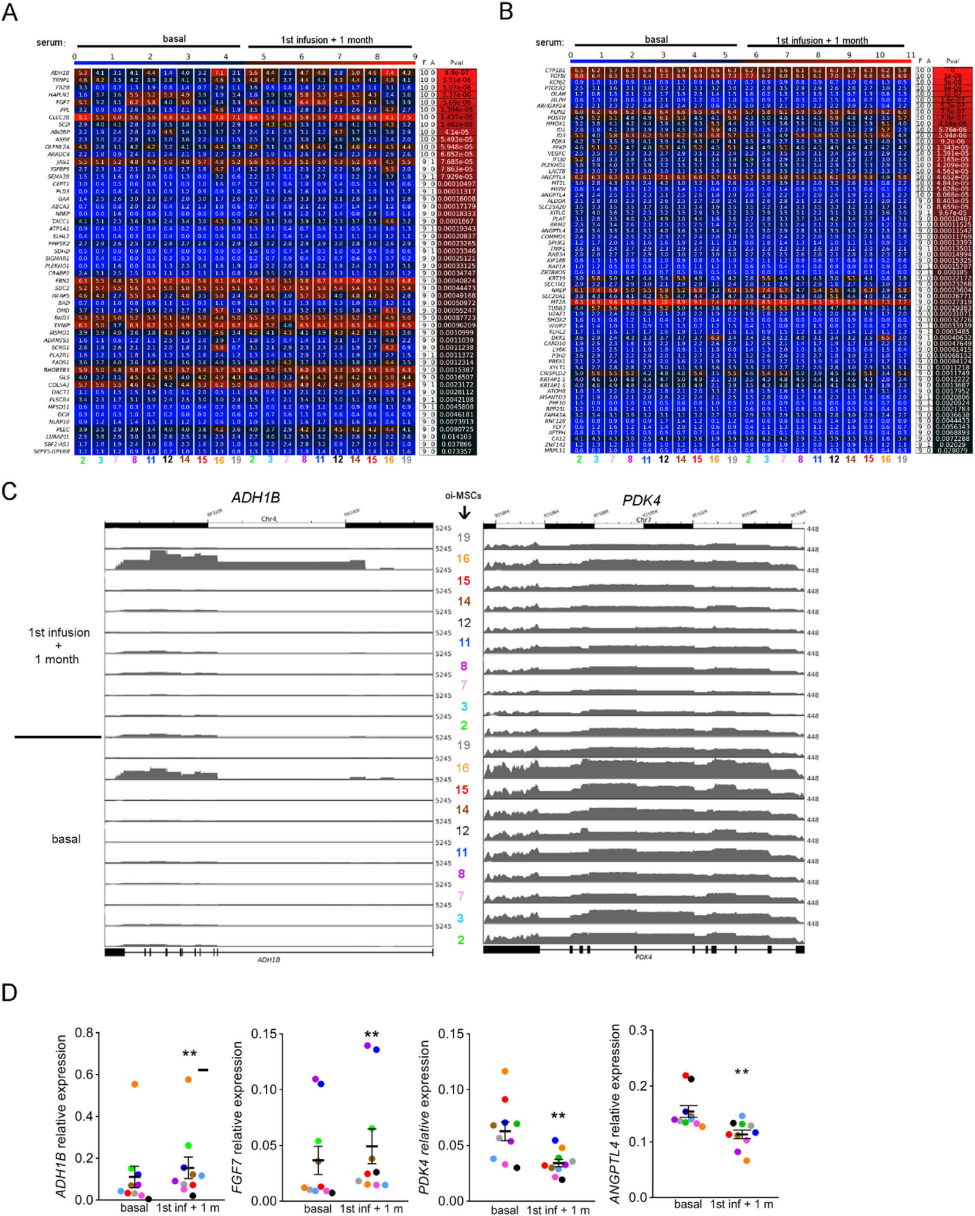
Transcriptome changes in OI MSCs in the presence of sera from P01 patient. (A and B) Heatmaps reflecting the significantly DEGs (rows) identified in the 10 OI MSCs (columns, each cell line identified by a number and a color) by RNAseq. The results were obtained comparing the data from OI MSCs cultured under osteogenic conditions (4 days) in the presence of the serum collected after the cell therapy (first infusion + 1 month) versus the serum collected before the treatment (basal). Numbers indicate the FPKM reads obtained from RNAseq for each DEG in each cell line. The columns “F” and “A” of the table denote how many votes are Favor and Against the analyzed condition, respectively. (C) Genome browser screenshot of RNAseq tracks at *ADH1B* and *PDK4* loci in the 10 OI MSCs lines analyzed, cultured under basal or 1 month after the first cell infusion serum. (D) Quantitative PCR validation of a subset of genes in the 10 OI MSCs lines. Each color represents an OI MSCs line. **P < .01

**FIGURE 7:**
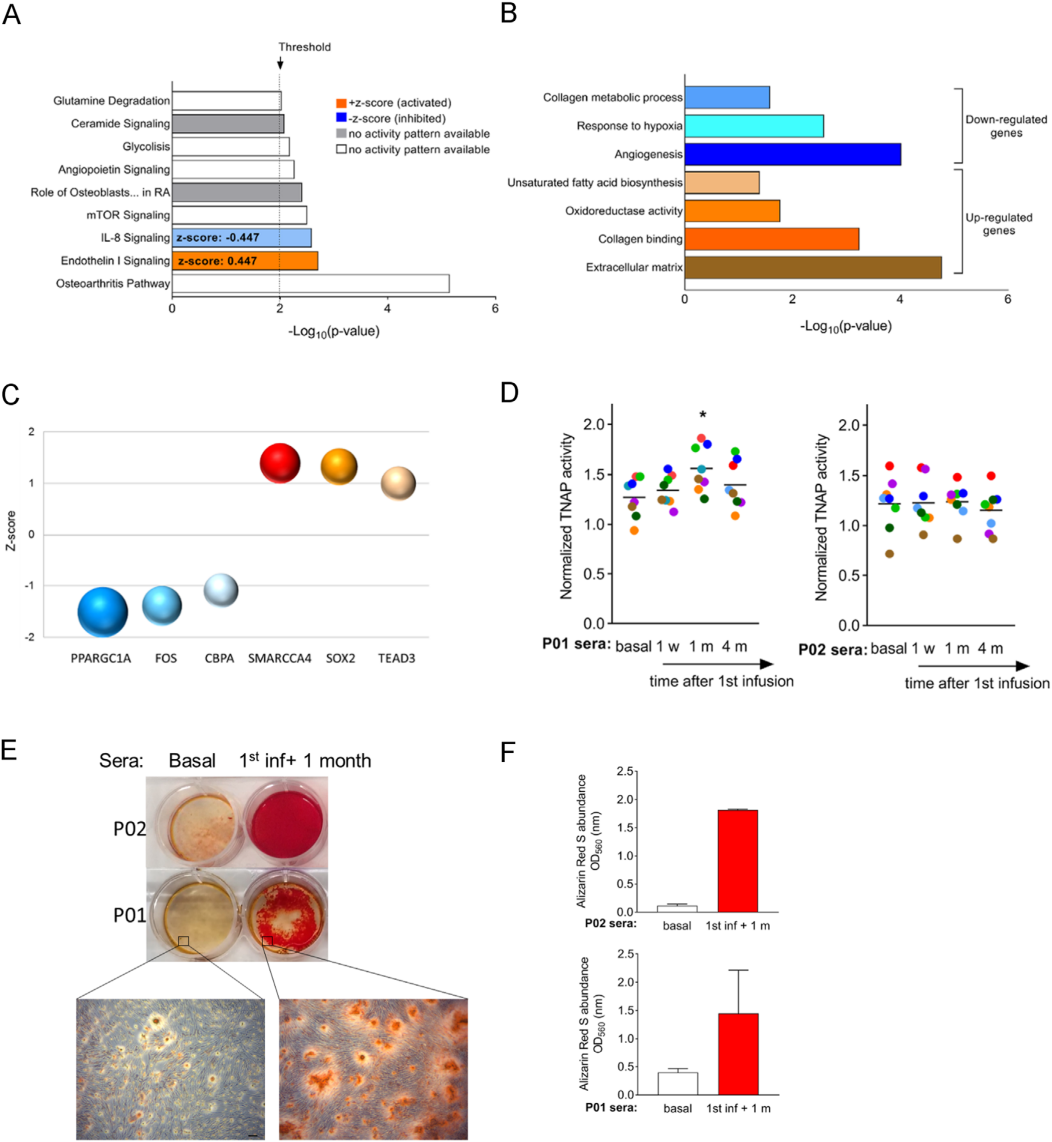
Assessment of the pro‐osteogenic potential of sera from TERCELOI patients. (A) IPA gene ontology enrichment analysis of the DEGs identified by RNAseq. (B) Significantly enriched biological processes identified by DAVID database. (C) Top six (three activated, *z*‐score > 1; three repressed, *z*‐score < 1) upstream regulators predicted by IPA to be responsible for the DEGs. Each transcription factor is represented by a sphere and plotted by its *z*‐score value. The *P*‐values for each transcription factor are graphically represented by different sphere sizes. (D) TNAP activity was measured (right graph) at day 4 of osteogenic induction in sera collected before (basal serum) and at different time points after the first cell infusion (1 w, 1 m, and 4 m). Each dot represents the TNAP activity value for an OI MSC cell line (n = 8, *P < .05). Horizontal lanes represent the mean of TNAP for each condition, two independent experiments). (E) Representative alizarin red S staining (ARS) staining of two OI MSCs lines cultured under osteogenic conditions (14 days) in the presence of TERCELOI sera collected before (basal) and 1 month after the first cell infusion. A field with a higher magnification shows the stained calcium deposits for each condition. Scale bar: 100 μm. (F) ARS quantification from triplicate wells of OI MSCs lines (n = 2) cultured with P01 or P02 sera. The mean and SEM was calculated from triplicates for each condition. Presented are representative data from two independent experiments

Having observed protein and microRNAs expression changes in patients’ sera after cell therapy, we wondered whether these sera could be pro‐osteogenic, which would be consistent with the improvements in bone phenotypes observed in patients after MSCs administration. To assess this possibility in vitro in the context of OI disease, we isolated MSCs from bone tissue samples from 10 OI pediatric patients (OI MSCs) who underwent correcting surgery. Isolated OI MSCs showed plastic adherence and a typical fibroblast spindle‐shaped morphology and a panel of cell surface markers that were in accordance with the immunophenotype defined for MSCs (Figure S1A). Their identity as progenitors of osteoblasts was confirmed by the extracellular matrix mineralization (increased calcium deposits), a feature of late osteogenesis,^46^ shown by OI MSCs after 21 days of osteogenic differentiation (Figure S1B). To study the early effects of sera from TERCELOI patients and elucidate if post‐cell therapy sera could have an effect on directing osteogenic fate of MSCs, we conducted a transcriptomic study based on RNA sequencing experiments (RNAseq). In this way, we compared the gene expression profiles from differentiated OI MSCs (10 OI MSCs lines) cultured in the presence of serum from P01 collected before the therapy (basal) versus the P01 serum collected 1 month (1 m) after the first cell infusion from the same patient. We considered that a gene was differentially expressed, when at least nine of the 10 cell lines analyzed showed consistent and significant (*P*‐value < .05) changes in its expression. Following these strict criteria, we found that when compared to basal serum, 1 m serum modulated the expression of 117 transcripts: 52 upregulated and 65 downregulated (Figure 6A‐C). These RNAseq results were validated by quantitative PCR: *ANGPTL4* and *PDK4*, classical targets of the adipogenic transcription factor PPARγ,^47^ showed a downregulation, whereas the metabolic enzyme *ADH1B* and the *FGF7* growth factor showed an upregulation upon cell culture of OI MSCs with 1 m serum (Figure 6D). Interestingly, analysis of the canonical pathways of genes dysregulated by IPA predicted an activation (positive *z*‐score) of “endothelin‐1 signaling” process, which is known to induce in vitro proliferation and differentiation of osteoprogenitors and in vivo trabecular bone formation (Figure 7A).^48^, ^49^, ^50^ “Glycolysis,” a major feature of osteogenic differentiation,^51^ was also identified as enriched. Conversely, the IL‐8 signaling pathway, which plays a central role in angiogenesis and inflammation,^52^ was identified as downregulated. Bone‐related categories such as “osteoarthritic pathway” and “role of osteoblasts in rheumatoid arthritis” were also identified as enriched among the DEGs (Figure 7A). DEGs were further analyzed with DAVID software (Figure 7B). MSCs undergoing osteogenic differentiation, even at early stages, are defined by the production of extracellular matrix proteins.^53^ Consistent with this fact, the most significantly enriched category identified by DAVID among the upregulated DEGs was “extracellular matrix” (*P*‐value: 1.69 × 10^−5^) (Figure 7B). Other categories related to “collagen binding,” “oxidoreductase activity,” and “unsaturated fatty acid biosynthesis” were also enriched (Figure 7B). Among the downregulated DEGs, categories related to “hypoxia,” “angiogenesis,” and “collagen metabolic process” were found to be significantly enriched (Figure 7B). To identify potential transcription factors driving the gene expression changes upon cell culture with serum obtained after cell therapy, the DEGs were further analyzed using the upstream regulator analysis in IPA. Strikingly, the top upstream regulators predicted to be inhibited were the pro‐adipogenic transcription factor PPARGC1A (*P*‐value: 2.45 × 10^−9^; *z*‐score: –1.507) (Figure 7C),^53^ CEBPA, also pro‐adipogenic (*P*‐value: 8.53 × 10^−5^; *z*‐score: –1.03), and FOS (*P*‐value: 4.35 × 10^−6^; *z*‐score: –1.03), identified previously as inhibited during osteogenesis of MSCs.^53^ On the contrary, the top transcription factor predicted as activated was the pro‐osteogenic SMARCA4 (*P*‐value: 2.89 × 10^−6^; *z*‐score: +1.394),^54^ followed by SOX2 (*P*‐value: 2.50 × 10^−5^; *z*‐score: 1.327) and TEAD3 (*P*‐value: 8.03 × 10^−5^; *z*‐score: +1.00). Collectively, these results suggest that P01 serum obtained 1 month (1 m) after the first cell infusion was able to activate an early pro‐osteogenic program in OI MSCs, by activating pro‐osteogenic transcription factors and inhibiting pro‐adipogenic ones.

We then evaluated the potential of TERCELOI sera on directing osteogenic fate of MSCs, by the determination of the activity of TNAP, an early marker of osteogenic differentiation.^46^ We cultured eight independent OI MSCs lines under osteogenic differentiation in the presence of basal serum, or sera collected after the first MSCs infusion in TERCELOI patients: 1 w, 1 m, and 4 m. TNAP activity was measured at day 4 of cell differentiation. Strikingly, 1 m serum from P01 significantly enhanced TNAP activity (Figure 7D, left), whereas sera from P02 did not affect TNAP activity in OI MSCs (Figure 7 D, right). Finally, we analyzed the potential of TERCELOI sera to induce the mineralization of OI MSCs lines. Strikingly, 1 m sera from both patients showed a great ability to induce mineralization of OI MSCs (enhanced calcium deposits), whereas basal serum did not (Figures 7E and 7F).

## 4 DISCUSSION

The transitory nature of the clinical benefits observed after MSCs therapy (one or two MSCs infusions) in OI patients is a major drawback of this treatment approach.^15^, ^16^, ^19^, ^20^ In TERCELOI, we have faced the previously described short‐term effects of MSCs therapy by performing repeated MSCs infusions in patients. To avoid subjecting the patients to alloimmunization after repeated exposure to non‐self MSCs, donor MSCs were HLA‐identical or histocompatible (five shared out of six HLA antigens). That level of histocompatibility was enough to accomplish the security of the five consecutive cell infusions in a period of 2.5 years with beneficial outcomes in both patients. Regarding the mechanism of action of cell therapy in OI, molecular and cellular studies have led to a change of paradigm, from the initially expected host tissue replacement of dead or injured cells by donor MSCs to the current paracrine hypothesis.^15^, ^16^ In fact, growing evidence from animal models of OI and other diseases suggests that MSCs exert their therapeutic effects, at least in part, via paracrine mechanisms.^21^, ^22^, ^55^, ^56^ In this way, the significance of our study is twofold: this is the first clinical trial testing the safety and effectivity of reiterative MSCs infusions in two nonimmunosuppressed OI pediatric patients, which at the same time, by studying the patients’ sera composition, begins to unravel the mode of action of MSCs in OI disease, a deficit in understanding previously recognized.^57^

Each enrolled OI patient showed a different severity: P01, with a very severe phenotype, and P02, with moderate clinical manifestations. The limited number of patients enrolled in TERCELOI was due to the highly restricted inclusion criteria required in the domestic pediatric patients affected by this rare bone disorder. After receiving five histocompatible MSCs infusions during a period of 2.5 years and followed an additional 2 years after the final cell infusion, neither of the patients suffered any adverse effects. Moreover, along the cell treatment (including the additional 2‐year follow‐up period), both patients showed durable improvements regarding the number of bone fractures and morphometric bone parameters, leading to a better HRQoL, which was noticed by patients and parents. In fact, patient‐reported outcome measures, such as the HRQoL, have been recently strongly recommended to be a highly valuable tool when assessing functional improvements in musculoskeletal disorders, especially those regarding cell‐based therapies.^35^ Regarding the clinical evaluation, despite the difficulty of extracting rigorous conclusions from what the families/parents experienced and related, in both cases it was remarkable that the patients dared to try new activities or games for the first time in their lives, feeling stronger, more optimistic, and confident besides with less fear of suffering a fracture. Of note, these improvements were especially noticeable after the two first MSCs infusions. This observation was supported in parallel with the study of bone tissue parameters: the major BMD increase as well as bone microstructure parameters such as TbSp, TbN, and QTS were observed in both patients after the two first infusions.

A major finding of our study, from a mechanistic point of view, is the existence of a systemic pro‐osteogenic response in OI patients as a consequence of MSCs therapy, which could be cause or consequence of the reported clinical improvements of patients. In that way, the cell therapy was able to elevate the serum levels of a therapeutic molecule recently shown to be increased in active healthy aging and known to be present in the developing mouse skeleton,^38^, ^39^ GPLD1. In fact, we detected a rise in GPLD1 levels as early as 7 days after the first cell infusion, especially in P01, who in addition showed this trend after the second infusion.

Our unprecedented patient's data about the mechanism of action of MSCs therapy suggest that the efficacy of the paracrine response induced by MSCs could depend on the severity of OI disease. Thus, the clinical benefits were more noticeable (reduction in number of fractures during and after the MSCs treatment) and with quite remarkable molecular findings (sera composition, miRNAs modulation, and increased TNAP activity) during and after cell therapy in P01, the most severely affected patient. Interestingly, basal serum from P01 showed a marked upregulation of the expression of almost all the miRNAs analyzed, which have been associated to osteoporotic bone fractures,^45^ but not P02. A number of recent studies have demonstrated a causative role for the dysregulation of certain miRNAs in bone disorders such as osteoporosis. MiR‐375 and miR‐214‐39, found to be significantly upregulated in basal serum from P01, have been shown to be upregulated in serum from osteoporosis patients with vertebral fractures.^44^, ^45^ Moreover, miR‐214‐3p was reported to be secreted in exosomes by osteoclasts and transferred to osteoblasts inhibiting osteoblastic bone formation.^44^ The overexpression of osteoporosis‐related miRNAs in P01 basal serum probably is a reflection of the most severe phenotype exhibited by this patient. Encouragingly, the reiterative MSCs infusions were able to modulate and finally repress this initial u‐regulation of miRNAs in P01. This interesting result suggests that at least at the miRNA level, the effect of MSCs infusion could not only be transitory but also cumulative because there is a gradual improvement with each cell infusion, the results of which last for at least 2 years after the final infusion. Moreover, our study reveals the potential enhanced expression of specific circulating miRNAs in the context of severe OI disease, a matter scarcely addressed in the literature.^58^ All in all, our data suggest that in severely affected patients, the upregulation of these miRNAs could be modulated and restored by continuous cell therapy.

Strikingly, when functionally challenged, sera from patients obtained after cell therapy showed pro‐osteogenic capabilities, which were more pronounced in the case of P01 serum. Thus, gene expression analysis showed a potential shift toward the osteogenic lineage (to the detriment of the adipogenesis process) when OI MSCs were cultured in the presence of serum collected after the first cell infusion. Consistent with this finding, TNAP activity, an early marker of osteogenesis, was enhanced in these cells. Strikingly, mineralization was also induced in OI MSCs cultured with sera from both TERCELOI patients collected after the cell therapy. Collectively, molecular and functional data obtained from sera analysis support the clinical benefits observed in OI pediatric patients treated with repeated infusions of HLA‐matched MSCs, indicating the activation of a pro‐osteogenic paracrine response as consequence of cell therapy.

How MSCs therapy modulates the levels of serum proteins and miRNAs in OI patients remains unknown, but it could be due to the paracrine properties of the MSCs. In fact, there are publications supporting this observation: the exosome secretion exerted by infused MSCs in a mouse model of lupus erythematosus was shown to be the underlying mechanism governing the improvement of the osteoporotic phenotypes in these mice.^55^ Moreover, the infusion of MSCs‐derived extracellular vesicles was shown to be responsible for improving bone growth in a mouse model of OI.^22^

Currently, an emerging concept is gaining attention, specifically the priming of MSCs to adapt their responses depending on the microenvironment they face.^59^ In TERCELOI, we have addressed the in vitro priming of MSCs with specific factors from the OI microenvironment (serum obtained before and after the cell therapy), finding that the osteogenic potential of MSCs can be enhanced with serum obtained after cell therapy. In this way, the in vivo bone healing potential of MSCs could be improved after being primed. Our results suggest that the greatest OI severity of P01 could be the underlying reason mediating his enhanced clinical and molecular outcomes. Thus, the most severe microenvironment in P01 (ie, upregulation of miRNAs) could be triggering a more efficient MSCs response in the interest of bone healing. In fact, a previous work demonstrated that in vitro MSCs priming with plasma coming from oim mice, a model of severe OI, led to increased chemotaxis and engraftment of transplanted MSCs in mice bones when compared to that shown by MSCs primed with control mice serum.^60^

## 5 CONCLUSIONS

In conclusion, and taking into account the limited number of patients, we can infer from the TERCELOI results that repeated MSCs therapy in pediatric patients is feasible and safe, supporting recent cell therapy attempts to other disorders based on sequential cell infusion.^61^, ^62^, ^63^ Regarding the effectiveness and mode of action of MSCs therapy, clinical improvements and noticeable pro‐osteogenic systemic response were detected as soon as the OI patients received the first cell infusion. Moreover, the clinical benefits persisted throughout the whole cell therapy treatment and up to the 2‐year follow‐up visit. However, some bone microstructure parameters, started worsening in the 2‐year follow‐up visit, suggesting repeated long‐term infusions are required to maintain the clinical benefits, especially in the more severe patients. In accordance with this observation, it is worth noting that after the 2‐year follow‐up visit, in the next couple of months P01 suffered three additional fractures.

For the future, detailed studies addressing the priming of MSCs in an OI specific context will elucidate if the most severe microenvironments (severe OI serum) could trigger different functions on MSCs. Secretomes from these primed MSCs, more easily obtained and free of the immune match requirements of MSCs, could be considered to be relevant surrogates of cell therapy (therefore democratizing the treatment), especially for chronic diseases such as OI.

## ETHICS APPROVAL AND CONSENT TO PARTICIPATE

The study was in accordance with the ethical standards formulated in the Helsinki Declaration and was approved by the Basque Ethics Committee for Clinical Research and the Spanish Agency of Medicines and Medical Devices (AEMPS).

## AVAILABILITY OF DATA AND MATERIAL

The data discussed in this publication have been deposited in NCBI's Gene Expression Omnibus (GEO)^64^ and are accessible through GEO Series accession number GSE157587 (https://www.ncbi.nlm.nih.gov/geo/query/acc.cgi?acc=GSE157587).

## CONFLICT OF INTEREST

The authors declare no conflict of interest.

## AUTHOR CONTRIBUTIONS

BG, MV, and CIR conceived the original idea and designed the clinical trial. CIR obtained administrative authorization for the clinical trial. IA was the sponsor of the clinical trial. BG, MV, IL, and GG were responsible for the clinical evaluations of patients. LM obtained the bone marrow samples. NO, AA, and AI performed HLA genotyping and analysis of immune activation. AI and CIR conceived and designed the experimental studies to elucidate the mechanism of action. AI carried out the in vitro cellular and molecular experiments. AMB provided the bone tissue samples from OI patients for OI MSCs isolation. BS provided information about the clinical course and follow‐up of the patients. DG and MJAB performed the RNAseq data analysis. AI, BG, and CIR wrote the manuscript. CIR obtained funding support. All authors approved the final version of the manuscript.

## Supporting information

Figure S1. OI MSCs characterization

Table S1. Most enriched categories among highly up‐regulated proteins in P01 serum collected 1 month after the first cell infusion
