# Supplementary figures and images for: Reiterative infusions of MSCs improve pediatric osteogenesis imperfecta eliciting a pro‐osteogenic paracrine response: TERCELOI clinical trial
Source: Clin Transl Med. 2021 Jan 13;11(1):e265. doi: 10.1002/ctm2.265 (PMC7805402; doi:10.1002/ctm2.265)

FIG. S1

A

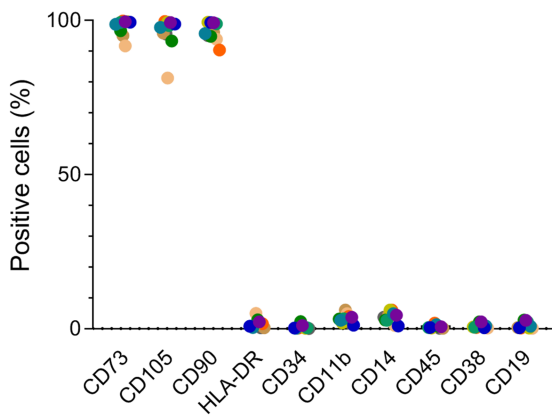

B

### oi-MSCs

OSTEOGENESIS (Alizarin Red; day 14)

ADIPOGENESIS (day 21)

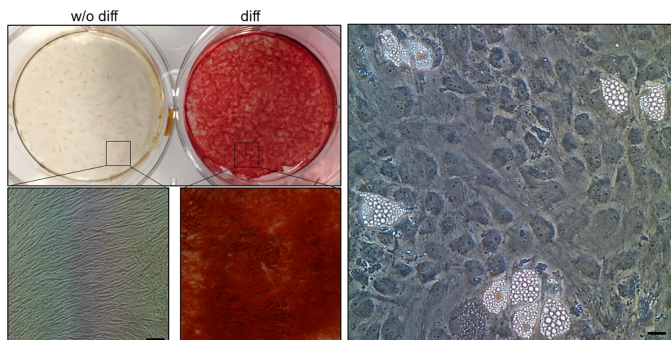

Supplement: Supplementary file 1 — Figure S1. OI MSCs characterization [file CTM2-11-e265-s001.pdf]
