# Supplementary material for: Reiterative infusions of MSCs improve pediatric osteogenesis imperfecta eliciting a pro‐osteogenic paracrine response: TERCELOI clinical trial
Source: Clin Transl Med. 2021 Jan 13;11(1):e265. doi: 10.1002/ctm2.265 (PMC7805402; doi:10.1002/ctm2.265)
Supplement: Supplementary file 2 — Table S1. Most enriched categories among highly up‐regulated proteins in P01 serum collected 1 month after the first cell infusion [file CTM2-11-e265-s002.docx]

**Supplementary Table I.** Most enriched categories among highly up-regulated proteins in P01 serum collected 1 month after the first cell infusion.

| **Gene Ontology term** | **p value** |
| --- | --- |
| GO:0006954~inflammatory response | 1.90E-33 |
| hsa04630:Jak-STAT signaling pathway | 2.52E-14 |
| GO:0001525~angiogenesis | 2.14E-13 |
| GO:0006935~chemotaxis | 2.86E-11 |
| GO:0030335~positive regulation of cell migration | 1.31E-08 |
| GO:0030509~BMP signaling pathway | 1.63E-06 |
| hsa04350:TGF-beta signaling pathway | 3.93E-06 |
| GO:0030574~collagen catabolic process | 4.13E-06 |
| hsa04151:PI3K-Akt signaling pathway | 5.62E-06 |
